# Plant Biomass Allocation-Regulated Nitrogen and Phosphorus Addition Effects on Ecosystem Carbon Fluxes of a Lucerne (*Medicago sativa* ssp. *sativa*) Plantation in the Loess Plateau

**DOI:** 10.3390/plants14040561

**Published:** 2025-02-12

**Authors:** Penghui Zhai, Rongrong Cheng, Zelin Gong, Jianhui Huang, Xuan Yang, Xiaolin Zhang, Xiang Zhao

**Affiliations:** 1 College of Grassland Science, Shanxi Agricultural University, Taigu, Jinzhong 030801, China; zhaipenghui@sxau.edu.cn (P.Z.); z20223246@stu.sxau.edu.cn (R.C.); z20213277@stu.sxau.edu.cn (Z.G.); yangxuan2019@sxau.edu.cn (X.Y.); 2 Shanxi Key Laboratory of Grassland Ecological Protection and Native Grass Germplasm Innovation, Taigu, Jinzhong 030801, China; 3 State Key Laboratory of Vegetation and Environmental Change, Institute of Botany, The Chinese Academy of Sciences, Beijing 100093, China; jhhuang@ibcas.ac.cn; 4 Key Laboratory of Model Innovation in Forage Production Efficiency, Ministry of Agriculture and Rural Affairs, Taigu, Jinzhong 030801, China

**Keywords:** lucerne, ecosystem respiration, gross ecosystem productivity, hay yield, net ecosystem productivity, nitrogen addition, phosphorus addition, plant biomass allocation

## Abstract

Nitrogen (N) and phosphorus (P) are key limiting factors for carbon (C) fluxes in artificial grasslands. The impact of their management on ecosystem C fluxes, including net ecosystem productivity (NEP), ecosystem respiration (ER), and gross ecosystem productivity (GEP) in the Loess Plateau is unclear. An experiment was conducted to study changes in these C fluxes with varying N (0, 5, 10, 15, and 20 g N m^−2^) and P (0 and 10 g P m^−2^) additions from 2022 to 2023 in a lucerne plantation. Results showed that N addition positively influenced NEP and GEP in the first year after planting with N addition at the rate of 10 g N m^−2^ was optimal for C assimilation, but it had negligible effect on ER in both two years in the studied lucerne (*Medicago sativa* ssp. *sativa*) plantation. Phosphorus addition significantly increased ER and stimulated GEP, resulting in an increasing effect on NEP only at the early stage after planting. The addition of N and P enhanced soil N and P availability and further improved the leaf chemical stoichiometry characteristics, leading to changes in biomass distribution. The more belowground biomass under N addition and more aboveground production under P addition resulted in different responses of ecosystem C fluxes to N and P addition. The results suggest that the effects of N and P fertilization management on the ecosystem C cycle may be largely dependent on the distribution of above- and belowground plant biomass in the artificial grassland ecosystem.

## 1. Introduction

The Loess Plateau is the world’s largest loess region and is characterized by extremely low mineral and nutrient availability. The ecosystems in the Loess Plateau are among the most vulnerable to excessive desertification [1,2]. This region is also a major hub for lucerne (*Medicago sativa* ssp. *sativa*) cultivation, an important forage resource that is a nitrogen-fixing plant known for its high nutritional value, high yield, and strong resistance to stress in temperate zones [3]. A previous study estimated that seeding legume forage could potentially increase soil organic carbon (C) storage by 147 million tons of CO_2_ equivalents per year in global pastures [4]. Lucerne, the world’s largest legume forage [5], has a strong C sink capacity [6], achieving a net ecosystem production of around 980 g CO_2_ m^−2^ per year [7]. Fertilization is the primary strategy to enhance forage quality and yield [8], and previous studies have shown that nitrogen (N) and phosphorus (P) limitations could affect terrestrial C uptake [9,10]. However, the regulation of lucerne grassland ecosystem C fluxes by N and P availability remains unclear.

Nitrogen is a crucial component of plant metabolites, which are closely related to leaf C exchange [11] and further impact ecosystem C cycle processes. Previous studies have shown that N fertilization significantly affects soil N availability and leaf N concentration, which are generally positively correlated with plant photosynthesis and growth, influence respiration, and therefore further affect ecosystem C cycles [12,13]. However, inconsistent results have also been reported, indicating that N decreased shoot N concentration [2], which is strongly related to biomass allocation [14,15], potentially leading to changes in the energy balance and CO_2_ production [16]. In addition, N has shown a threshold effect on the ecosystem C exchange in a meadow steppe [17]. Therefore, there might be an optimal amount of N addition for the ecosystem C cycle, a factor that remains largely unexplored in lucerne plantations.

Phosphorous is another important limiting factor for lucerne growth. It plays a direct role in many metabolic processes of plants, including photosynthesis and respiration [18]. However, it is easily lost from the field due to the lucerne harvest, leading to its exacerbated deficiency. Thus, P fertilization is an effective strategy to alleviate P limitation and maintain sustainability under continuous hay harvest of lucerne plantations, especially in the Loess Plateau [19,20]. Previous studies have shown that P addition can not only enhance soil fertility and improve plant P uptake and N absorption, thereby enhancing leaf physiological properties [21], but also improve N fixation of root nodules and increase lucerne productivity with plant biomass allocation to different organs [22,23]. As a result, the responses of the above variables may lead to changes in the ecosystem C cycling processes in lucerne plantations when facing P fertilization in this study area. In addition, P fertilization is typically used as a base fertilizer for a single time, resulting in rapid P fixation in soil and limited absorption by lucerne plants [24]. Therefore, it is necessary to increase P application frequency to improve its availability for plant growth, and more studies are needed to explore how P fertilization affects lucerne grassland ecosystem C fluxes. Moreover, how the increase in P application combined with N addition interactively affects ecosystem C exchanges in the lucerne plantation is far from clear in the Loess Plateau.

In summary, this study aimed to determine the responses of ecosystem C fluxes to N and P addition and to investigate the underlying mechanisms in a lucerne grassland plantation in the Loess Plateau, China. We measured the net ecosystem productivity (NEP), ecosystem respiration (ER), and gross ecosystem productivity (GEP) of a lucerne plantation, along with their associated biotic and abiotic factors. This was performed to address the following questions: (1) How do N and P additions affect the three ecosystem C fluxes differently? (2) Are the effects of N and P additions on ecosystem C fluxes regulated by plant biomass allocation?

## 2. Results

### 2.1. Abiotic and Biotic Factors

**Figure 1:**
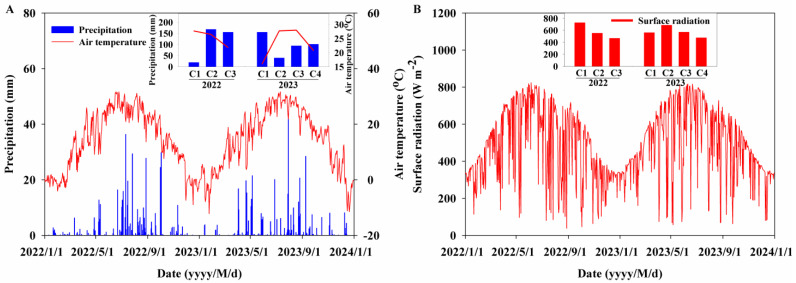
Daily values of (**A**) precipitation and average air temperature, (**B**) surface radiation from 2022 to 2023, with inset figures showing cumulative precipitation, average air temperature, and surface radiation from 8:00 to 12:00 across the first, second, and third cuts (C1, C2, and C3) in 2022, and across the first, second, third, and fourth cuts (C1, C2, C3, and C4) in 2023.

The precipitation amounts were 452.98 mm in 2022 and 444.83 mm in 2023, with rainfall accounting for 76.08% from June and 87.98% from March, both to the final cut of lucerne in 2022 and 2023, respectively (Figure 1A). The values of mean daily air temperature were 12.16 °C in 2022 and 12.39 °C in 2023, and the values of mean daily surface radiation were 184.97 W m^−2^ in 2022 and 188.23 W m^−2^ in 2023 (Figure 1). Additionally, the rainfall amount, the 8:00–12:00 air temperature, and 8:00–12:00 surface radiation were 19.72 mm, 27.73 °C, and 728.76 W m^−2^ across the first cut (C1), 168.78 mm, 26.52 °C, and 553.05 W m^−2^ across the second cut (C2), and 156.14 mm, 21.89 °C, and 468.71 W m^−2^ across the third cut (C3) of lucerne in 2022, respectively (Figure 1). The rainfall amount, the 8:00–12:00 air temperature, and 8:00–12:00 surface radiation were 155.69 mm, 16.05 °C, and 562.61 W m^−2^ across the C1, 39.92 mm, 27.78 °C, and 687.68 W m^−2^ across the C2, 94.32 mm, 28.03 °C, and 571.43 W m^−2^ across the C3, and 101.44 mm, 20.84 °C, and 476.57 W m^−2^ across the fourth cut (C4) of lucerne in 2023, respectively (Figure 1).

The additions of N and P altered abiotic factors across the three cuts of lucerne. Nitrogen addition increased soil NH_4_^+^–N at C2 and enhanced soil NO_3_^−^–N and AP at C3, respectively, in 2022, but it decreased soil NH_4_^+^–N at C3 and C4 and lowered soil NO_3_^−^–N at C2, respectively, in 2023 (Table S1). Phosphorus addition stimulated soil NH_4_^+^–N at C2 and increased AP at each cut in 2022 and 2023 (Table S1).

**Figure 2:**
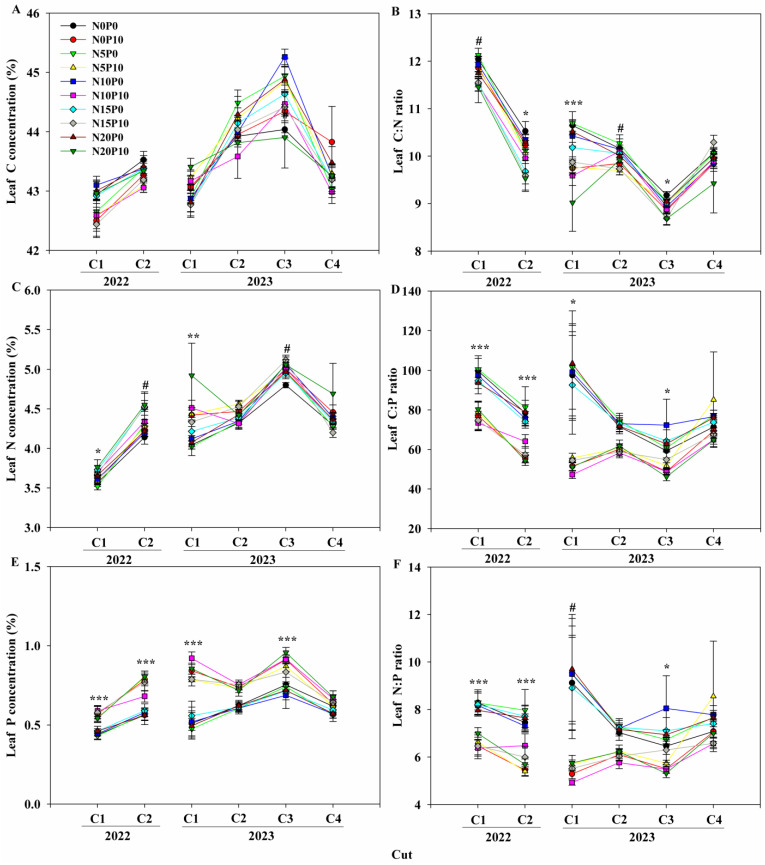
Dynamics of (**A**) leaf carbon (C) concentration, (**B**) leaf carbon–nitrogen (LC:N) ratio, (**C**) leaf nitrogen (N) concentration, (**D**) leaf carbon–phosphorous (LC:P) ratio, (**E**) leaf phosphorous (P) concentration, and (**F**) leaf nitrogen–phosphorous (LN:P) ratio of lucerne (*Medicago sativa* ssp. *sativa*) at the first and second cuts (C1 and C2) in 2022, and at the first, second, third, and fourth cuts (C1, C2, C3, and C4) in 2023. #, *, **, and *** represent the significant with *p* < 0.1, *p* < 0.05, *p* < 0.01, and *p* < 0.001 among all treatments, respectively.

**Figure 3:**
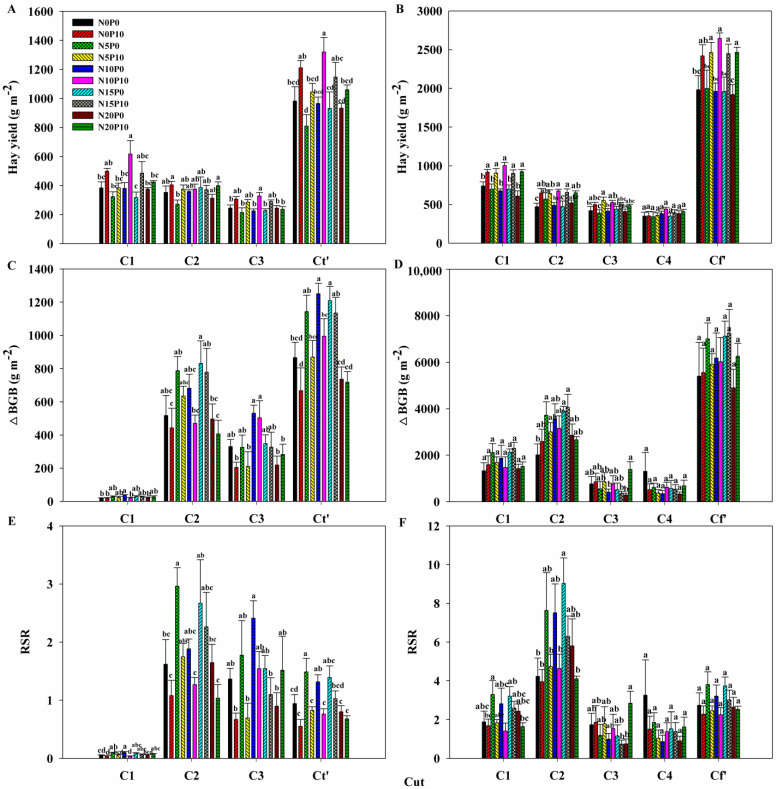
Mean values of (**A**,**B**) hay yield, (**C**,**D**) belowground biomass gain (ΔBGB), and (**E**,**F**) changes in root-to-shoot ratio (ΔRSR) at the first, second, third, and total three cuts (C1, C2, C3, and Ct’) of lucerne in 2022 and at the first, second, third, fourth and total four cuts (C1, C2, C3, C4, and Cf’) in 2023. Treatments: N0P0, no nitrogen (N) and phosphorus (P) addition; N0P10, no N addition but 10 g P m^−2^ addition; N5P0, 5 g N m^−2^ without P addition; N5P10, 5 g N m^−2^ with 10 g P m^−2^ addition; N10P0, 10 g N m^−2^ without P addition; N10P10, 10 g N m^−2^ with 10 g P m^−2^ addition; N15P0, 15 g N m^−2^ without P addition; N15P10, 15 g N m^−2^ with 10 g P m^−2^ addition; N20P0, 20 g N m^−2^ without P addition; N20P10, 20 g N m^−2^ with 10 g P m^−2^ addition. Values represent the mean ± 1SE (*n* = 5). Different lowercase letters in the bar chart indicate significant differences among all treatments at *p* < 0.05.

All the biotic factors that responded to N and P addition differed among the various cuts of lucerne. Nitrogen addition increased leaf N concentration but decreased the leaf C:N ratios at C1 and C2 in 2022 (Figure 2). Nitrogen addition significantly increased ΔBGB and RSR at a total of three cuts (Ct’), respectively, in 2022, while it had insignificant effects on hay yield, ΔBGB and RSR at a total of four cuts (Cf’) in 2023 (Figure 3, Table 1). In addition, N addition only increased ΔRSR at C1 and enhanced ΔBGB and ΔRSR at C2 and C3 in 2022, and it only enhanced ΔBGB and ΔRSR at C2 in 2023 (Figure 3, Table 1).

Phosphorus addition stimulated leaf P concentration but lowered the leaf C:N, C:P, and N:P ratios at each cut in both two years, except its insignificant effects on ratios of leaf ecological stoichiometric characteristics at C4 in 2023, while it increased leaf N concentration at C1, C2, and C3 in 2023 (Figure 2). Phosphorus addition increased hay yield but decreased ΔBGB and ΔRSR at Ct’ in 2022, and it enhanced hay yield but decreased ΔRSR at Cf’ in 2023 (Figure 3, Table 1). Additionally, it increased hay yield but lowered ΔRSR at each cut, and it decreased ΔBGB at C2 in 2022 (Figure 3, Table 1). It increased hay yield at C1, C2, and C3 but lowered ΔRSR at C1 and C2 in 2023 (Figure 3, Table 1).

There were no significant effects of N and P addition on leaf C concentration and above other variables, nor did they have interactive effects on all above variables with the exception of soil NH_4_^+^–N at C2 and ΔRSR at C1 in 2022 (Figure 2 and Figure 3, Table 1 and Table 2).

### 2.2. Effects of N and P Addition on Ecosystem C Fluxes 

**Figure 4:**
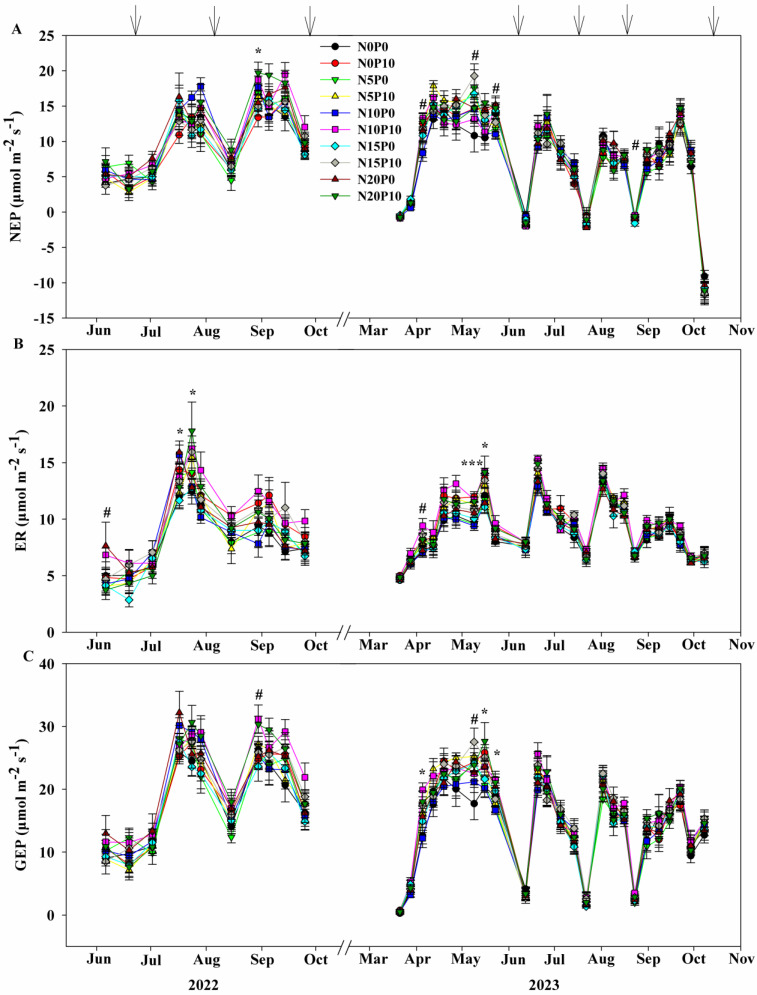
Seasonal dynamics of (**A**) net ecosystem productivity (NEP), (**B**) ecosystem respiration (ER), and (**C**) gross ecosystem productivity (GEP) during the growing season, which includes the first, second, and third cuts (C1, C2, and C3) in 2022, and the first, second, third, and fourth cuts (C1, C2, C3, and C4) in 2023. #, * and *** represent the significant with *p* < 0.1, *p* < 0.05 and *p* < 0.001 among all treatments, respectively.

In 2022 and 2023, N and P addition resulted in significant variations in the NEP, ER, and GEP of the lucerne plantation. The control treatment’s average values for NEP, ER, and GEP were 10.33 ± 0.43, 8.42 ± 0.66, and 18.74 ± 0.74 μmol m^−2^ s^−1^ across all three cuts (Ct), respectively, in 2022, and were 7.25 ± 0.40, 6.04 ± 0.53, and 14.02 ± 0.87 μmol m^−2^ s^−1^ across all four cuts (Cf), respectively, in 2023 (Figure 4).

Nitrogen addition significantly increased NEP and GEP, and these increases were significantly greater under 10 g N m^−2^ than that of 0 g N m^−2^, but it showed no significant difference with that of 15 and 20 g N m^−2^ across the Ct in 2022 (Figure 4, Table 2). Furthermore, N addition had no effect on ER across the Ct in 2022 and on the above three variables across the Cf in 2023 (Figure 4, Table 2). Additionally, N addition significantly increased GEP only across the C2, while it marginally enhanced NEP across the C2 and C3, respectively, in 2022, and it only marginally enhanced NEP across the C1 in 2023 (Figure 4, Table 2). Meanwhile, there was no significant effect of N addition on the above three processed across other cuts in 2022 and 2023 (Table 2).

The main effects of P addition on NEP, ER, and GEP significantly varied across all cuts (Table 2). Phosphorus addition significantly increased ER and marginally increased GEP across the Ct in 2022 and significantly increased ER and GEP across the Cf in 2023, but it had no effect on NEP across the above cuts in both 2022 and 2023 (Figure 4, Table 2). Phosphorus addition marginally increased NEP only across the C3 in 2022 and significantly stimulated it across the C1 in 2023 (Figure 4, Table 2). It significantly increased ER across the C2 and C3 in 2022 and stimulated it across the C1, C2, and C3 in 2023 (Figure 4, Table 2). It enhanced GEP across the C3 in 2022 and increased it across the C1 and C2 in 2023 (Figure 4, Table 2). However, P addition had no significant effect on the three ecosystem C fluxes across other cuts in both two years (Table 2).

The interactive effect of N addition and P addition was significant only on ER across the C1 in 2022, but it was insignificant across each cut, Ct, and Cf in two years. The three ecosystem C processes changed significantly over time in both two years except for C1 in 2022, while N addition effects only significantly changed on NEP and GEP over time across C1 in 2023. And P addition effects significantly changed both on ER and GEP over time across C2 and Ct in 2022, on ER across C1, C2, and Cf, and on GEP across C1 in 2023. In contrast, the interaction effect between N addition and P addition was only significant on ER over time at C2 in 2022 (Table 2).

### 2.3. Relationships of Ecosystem C Fluxes with Biotic and Abiotic Factors

**Figure 5:**
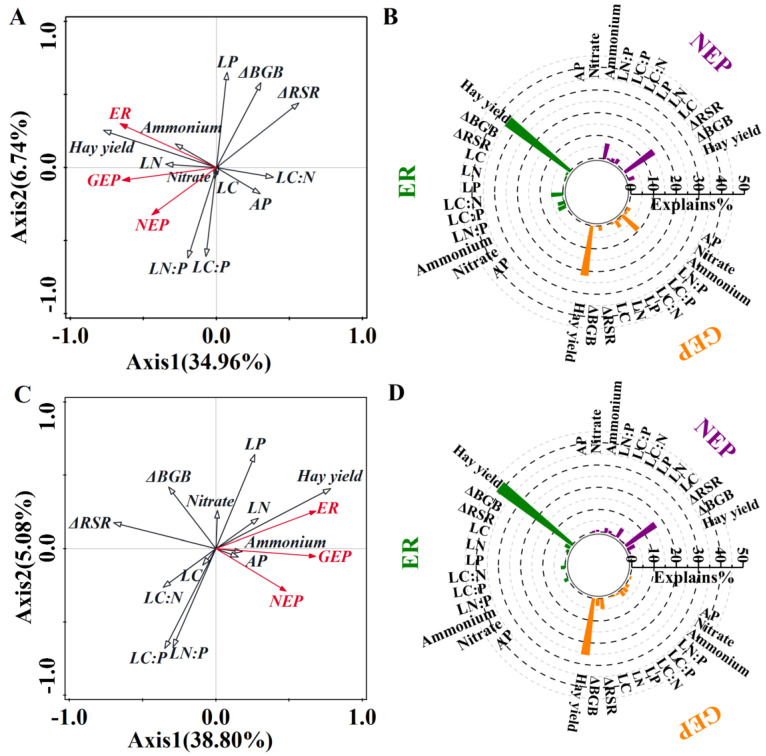
Redundancy analysis (RDA) on the response ratio of net ecosystem productivity (NEP), ecosystem respiration (ER), and gross ecosystem productivity (GEP) constrained by the response ratio of relevant biotic and abiotic factors to (**A**) nitrogen addition and (**C**) phosphorous addition (left panels). It also presents the percentage of variation by relevant biotic and abiotic factors to (**B**) nitrogen (N) addition and (**D**) phosphorous (P) addition (right panels).

Redundancy analysis (RDA) showed that the biotic and abiotic indicators explained 34.96% and 6.74% of the variations in ecosystem C fluxes due to N addition (Figure 5A), respectively, and explained 38.8% and 5.08% of the variations in ecosystem C fluxes due to P addition on the first and second axes, respectively (Figure 5C). However, the variations of biotic and abiotic indicators inconsistently impacted NEP, ER, and GEP. The leaf P concentration, leaf N:P ratio, hay yield, and ΔRSR were the major drivers of NEP, while soil NH_4_^+^–N, leaf physiological characteristics including leaf C:N and N:P ratio, and hay yield had a larger projection on ER and leaf C:N and N:P ratio, hay yield and ΔRSR had a greater positive projection on GEP due to N addition (Figure 5B). In addition, leaf P concentration and ΔRSR had a stronger positive projection on NEP, whereas only hay yield had greater positive correlations with ER, and hay yield and ΔRSR had a larger projection on GEP (Figure 5D).

**Figure 6:**
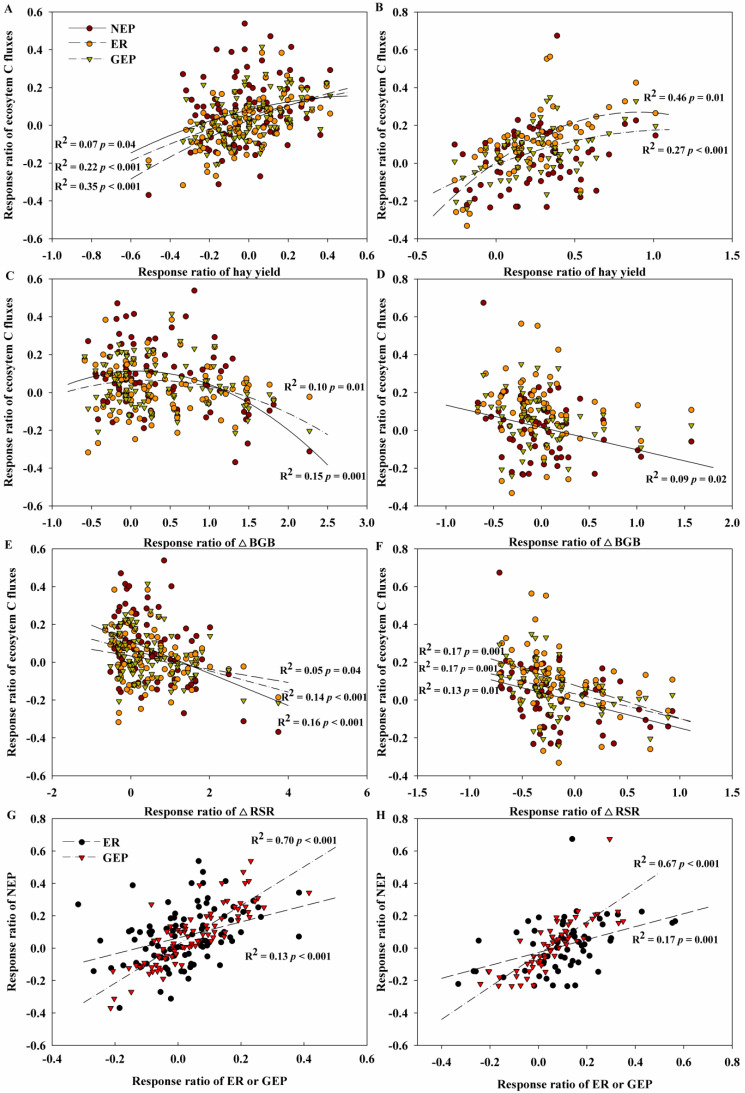
Relationships of the response ratios of ecosystem carbon (C) fluxes, including net ecosystem productivity (NEP), ecosystem respiration (ER), and gross ecosystem photosynthesis (GEP), with the response ratios of (**A**,**B**) hay yield, (**C**,**D**) belowground biomass gain (ΔBGB) and (**E**,**F**) change in root-to-shoot ratio (ΔRSR), and (**G**,**H**) response ratios of NEP with ER and GEP to nitrogen (N) addition (left panels) and to phosphorous (P) addition (right panels).

Further analysis revealed that the response ratio of ecosystem C fluxes, including NEP, ER, and GEP to N and P addition, exhibited quadratic relationships with that of hay yield, showing an initial increase followed by a decrease across all the cuts of lucerne except for NEP to P addition (Figure 6A,B). The response ratios of NEP and GEP to N addition demonstrated quadratic correlations with that of ΔBGB, while that of NEP to P addition showed a linear decreasing relationship with that of ΔBGB (Figure 6C,D). The response ratio of NEP, ER, and GEP to N and P addition showed a linear declining relationship with that of ΔRSR (Figure 6E,F). Additionally, the response ratio of NEP to N and P addition was both significantly positively correlated with GEP and ER across all the cuts of lucerne, with the correlation coefficients between NEP and GEP being higher than that between NEP and ER (Figure 6G,H).

**Figure 7:**
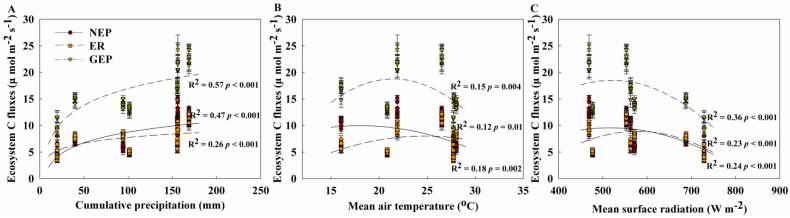
Relationships of ecosystem C fluxes, including net ecosystem productivity (NEP), ecosystem respiration (ER), and gross ecosystem productivity (GEP) with (**A**) cumulative precipitation, (**B**) mean air temperature from 8:00 to 12:00, (**C**) mean surface radiation from 8:00 to 12:00 across all the cuts of lucerne from 2022 to 2023.

NEP, ER, and GEP demonstrated significantly positive relationships with precipitation, air temperature, and surface horizontal radiation across the three cuts of lucerne (Figure 7). The NEP, ER, and GEP presented significant logarithmic relationships with precipitation across all cuts of lucerne (Figure 7A), and they exhibited quadratic relationships with air temperature and surface horizontal radiation, showing an initial increase followed by a decreasing trend (Figure 7B,C).

## 3. Discussion

### 3.1. Nitrogen and Phosphorus Addition Impacted the Three Ecosystem C Fluxes Differently

Nitrogen and P availability limitation in soil impacted plant growth to influence ecosystem C fluxes [25,26], particularly in the newly planted stage, although lucerne was less reliant on N fertilization due to the N fixation function of rhizobia [27]. There were increases in responses to N addition for NEP and GEP but not for ER, indicating that N is a crucial determinant for ecosystem C sequestration but showed minimal overall effect on C emission with an optimal addition amount at around 10 g N m^−2^ at the early stage of lucerne planting in this study. Phosphorus addition significantly increased ER and marginally increased GEP but insignificantly affected NEP in the study area, corroborating that P addition benefits the ecosystem C cycling [21], suggesting the importance of P fertilization in alleviating soil P limitation. Additionally, NEP was closely correlated with GEP but not with ER, suggesting the two opposing ecosystem C cycling processes are changing asynchronously, with C accumulation being impacted more by the photosynthesis process than the respiration process.

Remarkably, divergent responses of NEP, ER, and GEP to N and P addition in the early growth stage of lucerne were possibly controlled by different factors. On the one hand, soil NH_4_^+^–N, NO_3_—N, and P availability values under control treatment were around or lower than the background values, and they increased under N and P additions, suggesting that the more plant N and P absorption under suitable environmental conditions [28]. This then resulted in the greater enhancement of leaf N and P concentration, which would further decrease the leaf C:N, LC:P, and LN:P ratios of lucerne, reflecting greater leaf photosynthetic capacity and plant growth rate [29,30], and finally leading to higher ecosystem C assimilation. On the other hand, N and P addition stimulated phosphatase activity [31] to increase soil AP, with the increase in soil N availability, which could determine plant growth, including shoot growth, root growth, and their distribution [32,33,34], and subsequently influenced the three ecosystem C flux processes. In this study, N addition increased plant growth on ΔBGB but had no effect on hay yield, resulting in greater ΔRSR to enhance more on GEP than ER, leading to the increase in NEP. While P addition also increased plant growth of hay yields but lowered ΔBGB, resulting in lesser ΔRSR to stimulate ER more than GEP, causing no change in NEP. A previous study reported that P addition affected ecosystem C fluxes by stimulating soil respiration through decreasing RSR [35]. This study suggests that N and P addition induced potential changes in plant vertical structure with divergent biomass allocation to influence ecosystem C fluxes.

In addition, it was also possibly due to the weak N-fixing ability of the papillary bacteria of the *Rhizobium*, *Bradyrhizobium*, and *Sinorhizobium* groups living in symbiosis with legumes in the early stages, while N addition effect on ecosystem C fluxes was insignificant with the enhancement of their N fixation ability at the second planting year. Additionally, P addition effects on the three above C fluxes were increasing in the first year while decreasing in the second year after planting. Our results indicate that both N and P additions positively affect ecosystem C fluxes in the early stages after planting of lucerne, with their effects diminished over time.

### 3.2. Regulative Effects of Above- and Belowground Biomass Allocation on Ecosystem C Fluxes Under N and P Addition

Plant biomass allocation, including changes in the distribution of aboveground biomass of hay yield and belowground biomass, is crucial to understanding ecosystem functions such as material cycles [36]. Previous studies have shown that plant biomass allocation changes in response to potential environmental scenarios [37] and is regulated by adjusting the plant’s internal C-N status [38]. In this study, increasing the availability of N and P in soils enhanced their uptake by plants, thereby altering the allocation of above- and belowground biomass. The induced changes in the biomass distribution to above- and belowground influenced by changed leaf N and P concentrations and their ratios, which represented the root absorption capacity [30], further affected ecosystem C fluxes. The theoretical logic is that the N addition can increase soil N availability, improve leaf physiological characteristics, and subsequently cause more carbohydrate distribution to roots to maximize nutrient uptake. Previous meta-analyses have also found that N addition significantly increased RSR on a global scale due to its positive effect on total root biomass, with plants investing more in coarse roots to improve nutrient absorption ability rather than fine roots [39,40]. While increasing P supply caused a higher dry matter and shoot growth [41], it decreased ΔRSR, causing more biomass allocation to the aboveground, which is beneficial to capture more light resources [42,43,44]. In summary, lucerne is a perennial leguminous forage with a strong root system that distributes deep into the soil profile due to its strong tillering ability, quickly forming dense plant clusters, causing this discrepancy in the responses to N and P addition. Our results suggest that the root biomass of lucerne is more sensitive to N addition but less sensitive to P addition than the hay yield, resulting in unbalanced changes in plant biomass allocation due to their different resource capture capacities, leading to divergent changes among different ecosystem C fluxes.

Previous studies indicated that N and P exhibited extremely different properties in their biogeochemical cycles, with N showing stronger mobility than P in the soil, and declining P availability during long-term soil development [45] and is strongly precipitated by soil particles [26], resulting in the decreased aboveground P concentration [46]. Legumes require high P applications for greater crop productivity [47]. However, a large amount of N and P in the soil is removed through lucerne cut, leading to a limiting state for N and other nutrients, despite lucerne’s ability to obtain N through biological N fixation with its symbiotic rhizobia [48,49]. A previous study showed that biological N fixation can only provide about half of the total N required in the year of planting [50]. Our results clearly showed that both N and P applications had facilitative effects on ecosystem C fluxes, with the changing effects of N and P over time suggesting the importance of N and P in the early stages of lucerne plantation.

In addition, previous studies found that climate drivers affected plant biomass allocation [51], such as precipitation, air temperature, and solar radiation, which could regulate N effects on ecosystem C processes [28]. In this study, precipitation, air temperature, and solar radiation were also tightly correlated with ecosystem C fluxes, indicating that climate drivers play a key role in regulating ecosystem C fluxes in the lucerne plantations of the Loess Plateau. However, these results may change over time or with variations in fertilization amount and frequency, necessitating further studies to uncover the underlying mechanisms affecting ecosystem C fluxes with future fertilization management in lucerne plantations.

## 4. Materials and Methods

### 4.1. Site Description

The study site was situated in the National Regional Test Station of Grass Varieties, College of Grassland Science, Shanxi Agricultural University (37°25′ N, 112°23′ E, 799 m above sea level) in Shanxi province, a significant part of the Loess Plateau region. The climate is characterized by a continental temperate monsoon, with concurrent occurrences of precipitation and heat. The temperature difference between day and night is relatively large, and the average annual temperature ranges between 9.5 and 10.5 °C. The average annual precipitation is 458 mm, with the majority occurring from July to September. According to the International Standard for Soil Texture Classification, the soil texture is loam. The region has calcareous cinnamon soil [5]. The background concentrations of soil ammonium (NH_4_^+^–N), nitrate (NO_3_^−^–N), available P (AP), and organic matter (OM) are 2.05 mg kg^−1^ (categorized as low), 21.93 mg kg^−1^ (medium), 3.49 mg kg^−1^ (very low), and 26.04 g kg^−1^ (medium), respectively. The soil pH value is 7.97.

### 4.2. Experimental Design and Treatments

A field experiment was conducted from June to September 2022 and March to October 2023 in a lucerne (cv. WL354) plantation, which was artificially sown in August 2021. The sowing density was 18 kg km^−2^, with a row spacing of 30 cm and a depth of 3–5 cm. Weeds were manually removed throughout the experiment. The experiment employed a randomized block design, with 5 levels of N addition (0, 5, 10, 15, 20 g N m^−2^, respectively labeled as N0, N5, N10, N15, and N20) and combined with 2 levels of P addition (0, 10 g P m^−2^, respectively labeled as P0, P10). A total of 10 treatments were set up (N0P0, N0P10, N5P0, N5P10, N10P0, N10P10, N15P0, N15P10, N20P0, N20P10), with 5 replicates for each treatment, totaling 50 plots. Each plot covered an area of 20 m^2^ (4 m × 5 m), and the distance between adjacent plots was approximately 1 m. The form of N addition was urea, and the form of P addition was superphosphate. The fertilizations were evenly applied at the re-greening stage, after C1, C2, and C3 in 2022 and 2023, and after C4 in 2023.

### 4.3. Measurements in Ecosystem C Fluxes

Stainless steel frames (50 cm × 50 cm) were inserted about 8 cm into the soil and left 2 cm aboveground, approximately 1 m from the edge, to measure ecosystem C fluxes, including NEP, ER, and GEP. Measurements were conducted between 8:00 and 11:30 am every two weeks from June to September in 2022 and weekly from March to October in 2023. Ecosystem C fluxes were measured using an infrared gas analyzer (IRGA; LI–850, LI–COR Inc., Lincoln, NE, USA) connected to a transparent chamber. The transparent chamber (50 cm × 50 cm × 60 cm) had two fans at its top diagonal corners to evenly mix the air during measurement. It was necessary to fully ventilate the transparent chamber before measuring NEP and ER, with the entire measurement process lasting 80 s, and their values were calculated based on the recorded data. The difference between the two measurement processes was that a light-proof cloth cover should be placed around the transparent chamber when measuring ER. The GEP value was calculated as the sum of the NEP and ER values. The NEP, ER, and GEP values are calculated by Equations (1)–(3), according to a previous study [52]:

(1) $$NEP=-\frac{V\times Pav\times \left(1000-Wav\right)}{R\times S\times \left(Tav+273\right)}\times \frac{dc}{dt}$$

(2) $$ER=\frac{V\times Pav\times \left(1000-Wav\right)}{R\times S\times \left(Tav+273\right)}\times \frac{dc}{dt}\;$$

*GEP* = *NEP* + *ER* (3)

In the equations, *V* represents the volume of the measuring space (m^3^); *Pav*, *Wav*, and *Tav* represent the average values of atmospheric pressure (kPa), water vapor mole fraction (mmol mol^−1^), and temperature (°C) in the transparent chamber during measurement, respectively; *R* is the atmospheric constant of 8.314 J mol^−1^ K^−1^ under ideal conditions; *S* is the area (m^2^) of the stainless steel base; and *dc*/*dt* represents the slope of the least squares linear regression of CO_2_ concentration over time during the measurement period.

### 4.4. Soil and Plant Sampling

After harvesting lucerne, soil from 0 to 10 cm was collected from each plot, with about 10 g of soil weighed, extracted with 50 mL of 0.5 mol/L K_2_SO_4_, and then centrifuged for 1 h [53]. The concentrations of soil NH_4_^+^–N and NO_3_^−^–N were determined by a Flow Auto Analyzer (Continuous Flow Analysis—CFA, SEAL Auto Analyzer 3, Hamburg, Germany). Air-dried soil was extracted by 0.5 mol/L NaHCO_3_ and the molybdenum-antimony resistance colorimetric method [54], and then soil AP was determined by a UV/VIS spectrophotometer (UV–5800 PC, Shanghai, China).

Lucerne was harvested by strip-cut (50 cm × 50 cm) on 23 June 2022, 8 August 2022, 4 October 2022, 6 June 2023, 17 July 2023, 18 August 2023, and 12 October 2023. Leaving a 5 cm stubble at each cut, all plant samples were taken back and placed in an oven at 105 °C for 30 min and then oven-dried to a constant weight at 65 °C to determine the hay yield. The lucerne leaves were ground to a fine powder using a ball mill (Retsch MM 400; Retsch, Haan, Germany). Leaf C and N concentrations were determined using an Elemental Analyzer Vario Macro Cube model (Elementar Analysensysteme GmbH, Langenselbold, Germany). Leaf P concentration was determined using the Flow Auto Analyzer (Continuous Flow Analysis—CFA, SEAL Auto Analyzer 3, Hamburg, Germany). The leaf C:N ratio was calculated as the leaf C concentration divided by the leaf N concentration. The leaf C:P ratio was calculated as the leaf C concentration divided by the leaf P concentration. The leaf N:P ratio was calculated as the leaf N concentration divided by the leaf P concentration.

Simultaneously, soil was collected using an 8 cm diameter drill at a depth of 0–30 cm hole from the lucerne cut position two times repeated in each plot. The roots were collected after the soil was washed away and then dried in the oven to a constant weight to determine the belowground biomass. The belowground biomass gain (ΔBGB) was calculated as the growth amount before and after two cuts. The change in root-to-shoot ratio (ΔRSR) was calculated as the ΔBGB divided by the hay yield.

### 4.5. Statistical Analysis

Repeated-measures ANOVAs were utilized to identify the primary and interactive effects of sampling day, N addition, and P addition on NEP, ER, and GEP during each cut and across all the cuts during the growing season in 2022 and 2023, respectively. The post hoc test was performed with Duncan’s multiple range tests. Nonlinear regression analysis was employed to explore the relationships between the response ratio of ecosystem C fluxes and the response ratio of hay yield, ΔBGB, and ΔRSR to N and P addition, respectively. Linear regression was applied to analyze the relationships between the response ratios of NEP, ER, and GEP to N and P addition. Additionally, nonlinear regression analysis was used to explore the relationships of ecosystem C fluxes with cumulative precipitation, air temperature, and surface horizontal radiation. All the above statistical analyses were performed using SPSS 23.0 for Windows (SPSS Inc., Chicago, IL, USA). Redundancy analysis (RDA) was used to quantify the contributions of relevant biotic and abiotic indicators to NEP, ER, and GEP, and it was conducted by Canoco 5.0 software [53].

## 5. Conclusions

This study showed that N and P addition promoted ecosystem C assimilation at the early stage of lucerne planting, with their effects weakening following time in the Loess Plateau. The results from this study also suggest that the growth of belowground parts of lucerne is more sensitive to N addition than the aboveground parts, as indicated by a greater ΔRSR. This trend is reversed in the allocation of plant biomass under P addition, resulting in unbalanced changes in ecosystem C fluxes. The current study implies that ecosystem C fluxes in lucerne plantations may show strong uncertainty under different fertilization management practices due to unbalanced changes in the above- and belowground allocation of plant biomass, especially under future global climate change scenarios.

## Figures and Tables

**Table 1:** Results (*F*-values) of two-way ANOVAs on the effects of nitrogen (N) addition, phosphorus (P) addition, and their interactions on hay yield, belowground biomass gain (ΔBGB), and changes in root-to-shoot ratio (ΔRSR) at the first, second, and third cuts and total three cuts of lucerne (C1, C2, C3, and Ct’) in 2022, and at the first, second, third, fourth, and total four cuts (C1, C2, C3, C4, and Cf’) in 2023.

Variable	Treatment	2022				2023				
		C1	C2	C3	Ct’	C1	C2	C3	C4	Cf’
Hay yield	N	2.05	0.85	1.02	2.12	0.69	0.28	0.17	0.80	0.94
	P	13.88 ***	4.68 *	16.81 ***	21.08 ***	62.39 ***	22.67 ***	18.78 ***	1.28	32.05 ***
	N * P	1.07	0.92	1.61	0.52	0.97	0.48	0.72	0.15	0.22
ΔBGB	N	1.64	4.46 **	5.27 **	9.42 ***	1.75	3.58 **	0.53	0.62	1.26
	P	1.10	3.31 ^#^	1.08	7.69 **	0.07	0.14	2.72	0.17	0.002
	N * P	1.80	0.21	0.65	0.73	0.46	0.55	0.90	1.00	0.36
ΔRSR	N	2.31 ^#^	4.02 **	2.30 ^#^	5.19 **	1.70	2.80 *	0.60	0.84	1.29
	P	10.23 **	7.60 **	5.20 *	22.85 ***	8.56 **	8.43 **	1.73	0.37	6.08 *
	N * P	2.99 *	0.32	1.88	1.12	0.63	0.49	1.21	0.87	0.43

Note: ^#^, *, **, and *** represent the significance with *p* < 0.1, *p* < 0.05, *p* < 0.01, and *p* < 0.001 among all treatments, respectively.

**Table 2:** Results (*F*-values) of three-way ANOVAs examining the effects of the sampling day (Day), nitrogen (N) addition, phosphorus (P) addition, and their interactions on net ecosystem productivity (NEP), ecosystem respiration (ER), and gross ecosystem productivity (GEP) during the first, second, and third cuts and across all three cuts of lucerne (C1, C2, C3, and Ct), and the first, second, third, fourth, and across all four cuts (C1, C2, C3, C4, and Cf) in 2023.

Variable	Treatment	2022				2023				
		C1	C2	C3	Ct	C1	C2	C3	C4	Cf
NEP	N	0.26	2.10 ^#^	2.36 ^#^	4.24 **	2.37 ^#^	0.36	0.49	1.54	1.16
	P	1.40	0.95	2.80 ^#^	0.05	3.95 *	0.02	0.001	0.001	1.94
	Day	2.03	55.08 ***	117.87 ***	96.30 ***	273.14 ***	146.16 ***	195.09 ***	453.44 ***	294.21 ***
	N * P	0.78	0.31	0.73	0.36	0.98	0.63	1.09	0.89	0.25
	N * Day	0.64	0.78	0.98	0.73	1.66*	0.36	0.42	0.40	0.90
	P * Day	1.57	0.84	0.51	1.31	1.28	0.25	0.67	0.85	0.91
	N * P * Day	1.23	0.56	0.98	0.77	0.84	0.50	0.81	0.61	0.74
ER	N	1.15	1.02	0.39	0.64	0.22	0.03	0.76	0.15	0.17
	P	0.14	7.81 **	8.19 **	8.95 **	13.79 ***	7.19 **	2.75 ^#^	2.08	8.78 **
	Day	0.01	184.09 ***	13.57 ***	125.70 ***	341.47 ***	199.25 ***	412.57 ***	151.07 ***	278.85 ***
	N * P	3.89 **	0.29	0.65	0.98	0.55	0.21	0.06	0.41	0.24
	N * Day	0.42	1.08	0.69	0.77	0.39	0.60	0.82	0.67	0.56
	P * Day	0.46	8.34 ***	1.09	3.46 ***	5.81 ***	2.77 *	1.43	1.13	4.12 ***
	N * P * Day	1.10	2.12 *	0.62	1.23	0.70	0.72	0.63	0.56	0.72
GEP	N	0.50	2.72 *	1.01	2.59 *	0.45	0.28	0.60	0.56	0.21
	P	0.46	0.35	6.84 **	3.65 ^#^	11.56 **	3.09 ^#^	1.45	1.57	8.22 **
	Day	0.91	165.69 ***	113.78 ***	163.99 ***	474.74 ***	254.39 ***	489.55 ***	189.83 ***	308.66 ***
	N * P	1.19	0.24	0.44	0.42	0.83	0.20	1.03	0.14	0.23
	N * Day	0.56	0.68	0.76	0.55	1.46 ^#^	0.39	0.56	0.48	0.82
	P * Day	0.19	3.21 *	0.27	2.29 **	2.65 **	0.89	0.30	1.06	1.90
	N * P * Day	0.72	0.57	1.09	0.75	0.82	0.59	0.89	0.75	0.80

Note: ^#^, *, **, and *** represent the significance with *p* < 0.1, *p* < 0.05, *p* < 0.01, and *p* < 0.001 among all treatments, respectively.

## Data Availability

The original contributions presented in this study are included in the article/Supplementary Material. Further inquiries can be directed to the corresponding authors.

## Supplementary Materials

The following supporting informations can be downloaded at https://www.mdpi.com/article/10.3390/plants14040561/s1, Table S1: Mean values of soil ammonium (NH_4_^+^–N, mg kg^−1^), nitrate (NO_3_^−^–N, mg kg^−1^), and available phosphorus (AP, mg kg^−1^) at the first, second, and third cuts of lucerne (C1, C2, and C3) in 2022, and at the first, second, third, and fourth cuts (C1, C2, C3 and C4) in 2023; Table S2: Mean values of net ecosystem productivity (NEP), ecosystem respiration (ER), and gross ecosystem productivity (GEP) during the first, second, third cuts, and across the all three cuts of lucerne (C1, C2, C3, and Ct), and the first, second, third, fourth and across the all four cuts (C1, C2, C3, C4 and Cf) in 2023. Table S3: The equations of the lines and curves obtained in the regression analyses on Figure 6. Independent variable X, and dependent variable Y; Table S4: The equations of the lines and curves obtained in the regression analyses on Figure 7.
