# Supplementary material for: Plant Biomass Allocation-Regulated Nitrogen and Phosphorus Addition Effects on Ecosystem Carbon Fluxes of a Lucerne (Medicago sativa ssp. sativa) Plantation in the Loess Plateau
Source: Plants (Basel). 2025 Feb 12;14(4):561. doi: 10.3390/plants14040561 (PMC11859002; doi:10.3390/plants14040561)
Supplement: Supplementary file 1 [file plants-14-00561-s001.zip › plants-3437563-supplementary.pdf]

Table S1 Mean values of soil ammonium (NH_4^+–N, mg kg^{-1}), nitrate (NO_3^-–N, mg kg^{-1}), and available phosphorus (AP, mg kg^{-1}) at the first, second, and third cuts of lucerne (C1, C2, and C3) in 2022, and at the first, second, third, and fourth cuts (C1, C2, C3 and C4) in 2023.

Variable	Treatment	2022			2023			
		C1	C2	C3	C1	C2	C3	C4
NH_4^+–N	N0P0	2.45±0.39a	1.28±0.54b	3.04±0.61a	12.39±1.69a	7.40±0.95a	4.46±0.82a	14.62±1.27a
	N0P10	2.91±0.78a	2.80±0.54b	4.04±0.92a	12.53±0.61a	7.77±1.15a	2.18±0.70b	10.32±1.71ab
	N5P0	2.14±0.49a	2.49±0.59b	3.27±0.92a	12.02±0.94a	6.48±0.65a	2.08±0.68b	9.21±1.32ab
	N5P10	2.06±0.97a	2.13±0.55b	4.17±1.64a	12.42±1.02a	6.02±0.86a	1.72±0.43b	11.80±1.44ab
	N10P0	3.27±0.92a	2.41±0.74b	1.22±0.66a	10.40±1.28a	10.47±2.42a	2.20±0.54b	11.69±2.99ab
	N10P10	3.21±1.04a	2.94±0.38b	2.92±1.06a	12.83±1.59a	7.49±0.80a	1.88±0.35b	12.61±1.26ab
	N15P0	2.98±1.36a	2.28±0.35b	2.39±0.74a	10.35±2.08a	8.37±0.92a	3.14±0.50ab	10.61±1.17ab
	N15P10	3.69±1.30a	2.14±0.30b	1.80±0.91a	9.16±1.37a	9.94±1.47a	2.92±0.35ab	14.11±2.24a
	N20P0	3.02±0.97a	2.17±0.77b	2.38±0.93a	10.20±1.52a	9.27±2.67a	3.26±0.30ab	7.24±0.60b
	N20P10	1.17±0.40a	5.02±0.68a	1.78±0.97a	11.61±1.64a	8.20±1.59a	2.55±0.59b	7.92±1.43b
NO_3^-–N	N0P0	28.33±7.50a	29.25±6.89a	23.53±1.17ab	7.81±2.23a	20.85±6.04b	10.39±1.44abc	31.40±4.24a
	N0P10	26.68±4.98a	38.07±11.50a	28.14±1.59ab	6.63±1.08a	18.67±3.38b	11.38±1.00abc	29.99±6.93a
	N5P0	15.66±3.57a	27.31±6.78a	38.57±8.83ab	7.85±2.17a	14.30±2.01b	5.37±0.99c	30.21±7.13a
	N5P10	32.96±4.59a	27.85±8.43a	41.77±13.14a	5.13±0.84a	12.40±2.82b	12.78±1.05ab	25.66±5.34a
	N10P0	26.98±6.30a	27.34±5.69a	20.74±2.81b	9.23±0.90a	17.44±2.93b	13.86±3.24a	31.31±1.98a
	N10P10	28.69±9.15a	31.47±8.88a	26.80±3.56ab	9.69±1.09a	14.71±2.92b	10.96±2.91abc	36.10±7.18a
	N15P0	26.91±6.44a	29.85±7.58a	25.46±2.64ab	4.66±1.20a	14.33±4.08b	6.64±0.85bc	40.58±6.06a
	N15P10	34.22±5.98a	23.64±2.47a	28.71±3.81ab	6.44±1.14a	15.87±4.14b	12.43±2.42ab	32.16±4.32a
	N20P0	30.75±3.31a	20.98±2.91a	25.15±2.78ab	8.30±2.25a	13.31±2.70b	9.81±2.06abc	38.98±3.96a
	N20P10	29.61±6.01a	32.07±7.67a	35.38±9.02ab	9.34±3.28a	35.14±7.30a	10.89±2.24abc	31.32±4.78a
AP	N0P0	4.34±0.53b	4.91±0.96b	4.79±0.97b	3.44±0.33b	3.26±0.31b	2.99±0.40b	2.68±0.24c
	N0P10	10.71±0.62a	10.16±1.54a	11.68±1.60a	11.79±0.79a	11.74±0.53a	12.74±0.97a	11.16±0.31ab

N5P0	4.49±0.50b	5.15±0.46b	3.94±0.69b	3.18±0.54b	3.35±0.51b	3.47±0.81b	2.69±0.21c
N5P10	9.42±1.56a	10.34±0.82a	10.03±1.28a	14.06±1.00a	11.34±0.74a	11.47±0.56a	9.94±0.68b
N10P0	4.31±0.46b	4.06±0.72b	9.66±1.14a	3.12±0.39b	2.56±0.33b	3.26±0.57b	2.80±0.33c
N10P10	10.10±1.44a	10.04±0.79a	11.41±0.92a	11.28±0.57a	11.46±0.76a	11.74±0.57a	11.76±0.71a
N15P0	4.25±0.24b	5.18±0.97b	4.49±0.72b	4.02±0.06b	3.72±0.51b	3.06±0.33b	2.26±0.27c
N15P10	9.37±1.30a	10.98±1.79a	9.67±1.45a	11.47±0.49a	13.07±0.22a	13.54±0.75a	11.55±0.43a
N20P0	3.73±0.29b	3.17±0.76b	6.08±1.27b	3.39±0.14b	3.19±0.23b	5.91±2.99b	2.74±0.51c
N20P10	8.48±0.93a	10.64±1.76a	11.06±0.47a	11.77±0.38a	12.14±0.64a	12.13±0.50a	12.14±0.78a

Note: Values represent mean ± 1SE (n = 5). Different lowercase letters after the same column indicate significant differences at $P < 0.05$.

Table S2 Mean values of net ecosystem productivity (NEP), ecosystem respiration (ER), and gross ecosystem productivity (GEP) during the first, second, third cuts, and across the all three cuts of lucerne (C1, C2, C3, and Ct), and the first, second, third, fourth and across the all four cuts (C1, C2, C3, C4 and Cf) in 2023.

Variable	Treatment	2022				2023				
		C1	C2	C3	Ct	C1	C2	C3	C4	Cf
NEP	N0P0	4.37±0.84a	11.08±1.40ab	12.11±0.63b	10.33±0.43b	9.37±0.88c	7.54±0.42a	6.29±0.62a	4.87±0.34a	7.25±0.40a
	N0P10	4.54±1.29a	10.10±0.75b	11.64±0.77b	9.79±0.53b	11.10±0.56abc	6.83±0.57a	5.43±0.58a	4.80±0.27a	7.57±0.19a
	N5P0	6.65±1.25a	10.67±1.55ab	12.17±1.15b	10.62±0.90ab	10.74±0.78abc	6.54±0.42a	5.18±0.70a	4.41±0.30a	7.24±0.42a
	N5P10	3.80±0.82a	10.85±0.61ab	12.51±0.26ab	10.32±0.09b	11.41±0.38a	6.95±0.63a	5.75±0.75a	4.52±0.40a	7.68±0.25a
	N10P0	5.27±1.24a	13.28±0.40a	12.30±0.90b	11.38±0.48ab	9.48±0.57bc	7.57±0.44a	5.80±0.44a	4.85±0.33a	7.21±0.34a
	N10P10	5.09±0.39a	11.73±0.64ab	14.40±1.29ab	11.74±0.62a	10.48±0.43abc	7.17±0.41a	6.02±0.41a	5.13±0.36a	7.61±0.24a
	N15P0	5.10±0.92a	10.82±0.93ab	11.84±0.84b	10.24±0.52b	11.15±0.39ab	6.90±0.55a	5.78±0.42a	5.18±0.32a	7.77±0.21a
	N15P10	4.23±1.18a	10.96±1.14ab	12.68±0.52ab	10.52±0.62ab	11.38±0.24a	6.98±0.90a	6.75±0.64a	4.82±0.28a	7.92±0.29a
	N20P0	5.18±0.87a	12.83±0.59ab	13.29±1.37ab	11.65±0.71ab	11.24±0.43a	6.57±0.28a	6.30±0.52a	5.20±0.16a	7.82±0.16a
	N20P10	5.21±0.76a	12.14±0.67ab	15.19±0.45a	12.27±0.39a	11.00±0.36 abc	7.45±0.74a	5.39±0.54a	5.22±0.37a	7.77±0.24a
ER	N0P0	5.03±0.34ab	10.43±0.69b	8.16±0.91a	8.42±0.66b	5.40±0.62b	7.26±0.52a	7.87±0.48a	4.96±0.59a	6.04±0.53a
	N0P10	4.79±0.27ab	11.58±0.69ab	10.37±0.95a	9.80±0.66ab	6.94±0.50ab	8.24±0.64a	8.30±0.57a	4.74±0.35a	6.80±0.44a
	N5P0	4.58±0.37ab	11.13±0.65ab	8.28±0.66a	8.64±0.48b	5.57±0.58b	7.57±0.70a	8.03±0.70a	4.50±0.47a	6.07±0.57a
	N5P10	4.21±0.54b	11.58±0.33ab	8.99±0.79a	9.06±0.29ab	6.39±0.40ab	7.88±0.32a	8.45±0.37a	5.14±0.30a	6.67±0.29a
	N10P0	4.51±0.91ab	11.45±0.56ab	8.26±0.68a	8.74±0.49b	5.32±0.45b	7.31±0.30a	8.17±0.45a	4.69±0.52a	6.00±0.40a
	N10P10	6.47±0.66a	12.60±0.58a	10.75±0.74a	10.65±0.53a	7.48±0.64a	8.32±0.34a	8.89±0.32a	5.49±0.33a	7.32±0.39a
	N15P0	3.51±0.38b	10.44±0.49b	8.70±0.73a	8.39±0.57b	5.52±0.65b	7.28±0.49a	7.69±0.59a	4.71±0.48a	5.99±0.50a
	N15P10	5.32±1.11ab	12.03±0.55ab	9.88±0.70a	9.83±0.65ab	6.35±0.40ab	8.12±0.33a	8.30±0.34a	5.14±0.32a	6.68±0.30a
	N20P0	6.45±0.85a	11.39±0.46ab	8.68±0.70a	9.26±0.49ab	5.61±0.60b	7.49±0.28a	7.56±0.46a	4.88±0.49a	6.10±0.44a
	N20P10	4.05±0.45b	12.14±0.65ab	9.18±0.91a	9.32±0.59ab	6.66±0.55ab	8.14±0.29a	7.89±0.37a	5.20±0.33a	6.75±0.34a
GEP	N0P0	9.41±1.02a	21.51±1.20a	20.26±1.52b	18.74±0.74b	14.77±1.37b	14.80±0.62a	14.16±0.97a	12.43±0.85a	14.02±0.87a

N0P10	9.33±1.38a	21.68±1.09a	22.01±1.28ab	19.58±0.79ab	18.03±1.01a	15.07±0.84a	13.73±0.51a	12.68±0.55a	15.25±0.62a
N5P0	11.23±1.57a	21.80±2.09a	20.45±1.36ab	19.26±1.23b	16.31±1.18ab	14.11±0.77a	13.21±0.94a	12.27±0.97a	14.24±0.91a
N5P10	8.01±1.27a	22.43±0.63a	21.49±0.83ab	19.38±0.32ab	17.79±0.69a	14.84±0.65a	14.20±1.00a	12.81±0.56a	15.23±0.44a
N10P0	9.78±1.79a	24.73±0.38a	20.56±1.44ab	20.12±0.67ab	14.80±0.78b	14.88±0.59a	13.96±0.53a	12.68±0.86a	14.09±0.57a
N10P10	11.56±0.86a	24.32±1.03a	25.16±1.89a	22.38±1.05a	17.96±0.84a	15.49±0.57a	14.91±0.34a	13.89±0.52a	15.84±0.47a
N15P0	8.61±0.81a	21.26±1.13a	20.54±1.41ab	18.63±0.97b	16.67±0.64ab	14.17±1.00a	13.47±0.56a	12.96±0.91a	14.62±0.60a
N15P10	9.55±2.22a	22.99±1.53a	22.56±0.84ab	20.35±1.16ab	17.73±0.54ab	15.10±0.97a	15.06±0.55a	13.27±0.43a	15.53±0.46a
N20P0	11.63±1.09a	24.22±0.70a	21.98±2.02ab	20.91±1.17ab	16.85±0.87ab	14.07±0.43a	13.86±0.22a	13.04±0.68a	14.75±0.52a
N20P10	9.25±1.19a	24.28±0.96a	24.37±1.22ab	21.59±0.82ab	17.66±0.86ab	15.58±0.64a	13.29±0.52a	13.60±0.72a	15.41±0.44a

Note: Values represent mean ± 1SE (n = 5). Different lowercase letters after the same column indicate significant differences at $P < 0.05$.

Table S3 The equations of the lines and curves obtained in the regression analyses on Figure 6. Independent variable X, and dependent variable Y.

		Response ratios of NEP	Response ratios of ER	Response ratios of GEP
To N addition	Response ratios of hay yield	$Y=-0.212X^2+0.253X+0.082$	$Y=-0.166X^2+0.419X+0.027$	$Y=-0.126X^2+0.316X+0.048$
	Response ratios of ΔBGB	$Y=-0.086X^2+0.017X+0.112$		$Y=-0.059X^2+0.026X+0.062$
	Response ratios of ΔRSR	$Y=-0.085X+0.111$	$Y=-0.035X+0.033$	$Y=-0.056X+0.066$
	Response ratios of ER	$Y=0.495X+0.064$		
	Response ratios of GEP	$Y=1.199X+0.024$		
To P addition	Response ratios of hay yield		$Y=-0.323X^2+0.584X+0.006$	$Y=-0.138X^2+0.32X-0.007$
	Response ratios of ΔBGB	$Y=-0.118X+0.016$		
	Response ratios of ΔRSR	$Y=-0.142X-0.006$	$Y=-0.175X+0.078$	$Y=-0.132X+0.033$
	Response ratios of ER	$Y=0.399X-0.026$		
	Response ratios of GEP	$Y=1.006X-0.037$		

Table S4 The equations of the lines and curves obtained in the regression analyses on Figure 7.

	**NEP**	**ER**	**GEP**
Cumulative precipitation	$Y=2.875\ln(X)-4.507$	$Y=1.545\ln(X)+0.637$	$Y=4.563\ln(X)-4.06$
Mean air temperature	$Y=-0.033X^2+1.191X-0.781$	$Y=-0.033X^2+1.632X-12.165$	$Y=-0.106X^2+4.555X-30.162$
Mean surface radiation	$Y=-9.2E-05X^2+0.095X-14.971$	$Y=-0.00014X^2+0.156X-36.179$	$Y=-0.00017X^2+0.177X-27.562$
